# Uncovering the Rosetta Stone: Report from the First Annual Conference on Key Elements in Translating Stroke Therapeutics from Pre-Clinical to Clinical

**DOI:** 10.1007/s12975-018-0628-9

**Published:** 2018-04-10

**Authors:** Gregory J. Bix, Justin F. Fraser, William J. Mack, S. Thomas Carmichael, Miguel Perez-Pinzon, Halina Offner, Lauren Sansing, Francesca Bosetti, Cenk Ayata, Keith R. Pennypacker

**Affiliations:** 1 Center for Advanced Translational Stroke Science, University of Kentucky, Lexington, KY USA; 2 Sanders-Brown Center on Aging, University of Kentucky, Lexington, KY USA; 3 Department of Neurology, University of Kentucky, Lexington, KY USA; 4 Department of Neuroscience, University of Kentucky, Lexington, KY USA; 5 Department of Neurosurgery, University of Kentucky, Lexington, KY USA; 6 Department of Radiology, University of Kentucky, Lexington, KY USA; 7 Department of Neurosurgery, Keck School of Medicine, University of Southern California, California, Los Angeles USA; 8 Department of Neurology, David Geffen School of Medicine, University of California at Los Angeles, California, Los Angeles USA; 9 Department of Neurology, University of Miami Miller School of Medicine, Miami, Florida, USA; 10 Department of Neurology, Oregon Health & Science University, Portland, Oregon, USA; 11 Department of Anesthesiology, Oregon Health & Science University, Portland, Oregon, USA; 12 Perioperative Medicine, Oregon Health & Science University, Portland, Oregon, USA; 13 Department of Neurology, Yale University School of Medicine, New Haven, CT USA; 14 National Institute of Neurological Disorders and Stroke, National Institutes of Health, Bethesda, MD USA; 15 Department of Neurology, Harvard Medical School, Charlestown, MA USA; 16 Department of Radiology, Harvard Medical School, Charlestown, MA USA

**Keywords:** Clinical trials, Animal models, Reverse translation, Brain ischemia, Thrombectomy

## Abstract

The first annual Stroke Translational Research Advancement Workshop (STRAW), entitled “Uncovering the Rosetta Stone: Key Elements in Translating Stroke Therapeutics from Pre-Clinical to Clinical” was held at the University of Kentucky on October 4–5, 2017. This workshop was organized by the Center for Advanced Translational Stroke Science. The workshop consisted of 2 days of activities. These included three presentations establishing the areas of research in stroke therapeutics, discussing the routes for translation from bench to bedside, and identifying successes and failures in the field. On day 2, grant funding opportunities and goals for the National Institute for Neurological Diseases and Stroke were presented. In addition, the meeting also included break-out sessions designed to connect researchers in areas of stroke, and to foster potential collaborations. Finally, the meeting concluded with an open discussion among attendees led by a panel of experts.

## Introduction

Stroke remains a leading cause of death and disability worldwide [1–3]. While therapeutic research has yielded two vital acute interventional treatments for stroke, intravenous thrombolysis and mechanical thrombectomy [1, 4, 5], the vast majority of research has not yet translated into standardized and efficacious therapeutic strategies. Organized groups and professional societies have worked to overcome the failures of translation from bench to bedside. For example, the STAIR meetings have developed criteria for research models of stroke disease [6–9]. The adoption of these strategies has been relatively slow and requires continual emphasis and discussion [9]. Recently, the National Institute of Neurological Disorders and Stroke (NINDS) at the National Institute of Health (NIH) held a conference entitled the “Translational Stroke Research: Vision and Opportunities Workshop” [10]. This workshop was highly successful in bringing together researchers that could have a high impact on future stroke therapeutics [10]. In an effort to build upon these efforts, we organized the first annual Stroke Translational Research Advancement Workshop (STRAW), entitled “Uncovering the Rosetta Stone: Key Elements in Translating Stroke Therapeutics from Pre-Clinical to Clinical.” The workshop consisted of 2 days of activities. The first day included three presentations establishing the areas of research in stroke therapeutics, discussing the routes for translation from bench to bedside, and identifying successes and failures in the field. On day 2, grant funding opportunities and goals for the National Institute for Neurological Diseases and Stroke were presented. In addition, the meeting also included break-out sessions designed to connect researchers in areas of stroke and to foster potential collaborations. Finally, the meeting concluded with an open discussion among attendees led by a panel of experts. While the meeting was well-attended, we aim to publish the proceedings for the benefit of all engaged in stroke research.

## Conference Proceedings—October 4–5, 2017

### Leveling the Playing Field: Update on the Current Clinical Paradigm in Stroke and Opportunities of Translational Research (Presented by William J. Mack)

Engaging in true translational research for stroke therapeutics requires a working knowledge of the current state of treatment for acute stroke. For acute ischemic stroke, the most devastating subtype is emergent large vessel occlusion (ELVO); this involves acute occlusion of one of the main arteries of the Circle of Willis. During an ELVO, approximately 1.8 million neurons are lost per minute [11]. Standard treatment includes administration of intravenous (IV) tissue plasminogen activator (tPA), with a goal of emergency room arrival to infusion initiation (called the “Door to Needle” time) of less than 60 min [12]. However, IV tPA has varying efficacy depending on the stroke-affected artery (e.g., 31–44% recanalization of cases of a distal M2 branch of the middle cerebral artery (MCA), but only 4–8% recanalization of occlusions of the terminal internal carotid artery (ICA) [13, 14]). Given this issue, endovascular mechanical thrombectomy (MT) has provided an additional resource for acute recanalization of ELVO. While MT has expanded the window of intervention, time remains critical, as probability of good clinical outcome decreases rapidly over time [15]. In 2015, multiple clinical trials demonstrated the significant benefit in outcome when mechanical thrombectomy is used to treat ELVO [1, 5, 16, 17]. This has resulted in new guidelines for the use of mechanical thrombectomy to treat ischemic stroke [18–20]. Significantly, the most current clinical trials in this area, DEFUSE 3 and DAWN, suggest that the window of benefit for thrombectomy may extend to 16 or even 24 h from “last known normal,” respectively, in ischemic stroke patients who had a mismatch between the severity of clinical deficit and the infarct volume [21, 22].

Despite these advances in recanalization, there remain significant opportunities for therapeutic development in ischemic stroke. In a pooled analysis of the randomized clinical trials on thrombectomy conducted 2010–2014, only 26.9% of 633 patients that underwent thrombectomy achieved a modified Rankin score (mRS; a score that measures the degree of disability or dependence in the daily activities of people who have suffered a stroke or other neurological disability) of 0–1 (denoting minimal or no disability at 90 days) [23]. Though this represents a significant benefit over those who do not receive thrombectomy, it reflects a clear opportunity for further therapeutic development. Areas of most pressing need include advancements in time, access, and adjunctive neuroprotective strategies. Time is vital, as faster onset to treatment times have been associated with reduced mortality, reduced symptomatic intracranial hemorrhage, and increased independence after recanalization. Access to acute stroke care is also a major issue. In a study of geographic access, approximately 50% of the USA is within 60 min of ground access to the thrombectomy capable hospital, while 85% of the USA is within 60 min of air access [24]. Progress is advancing in terms of hospital systems; the majority of stroke patients in the USA are hospitalized in states where there are established or organizing stroke systems of care [25]. Codifying stroke systems of care will aid in rapid triage and transport, but time delays continue to exist and negatively impact outcome [26, 27]. From a translational therapeutics standpoint, these issues of time and access could be addressed, in part, through adjunctive therapies aimed at preserving tissue, enhancing collateral circulation, and protecting parenchyma from ischemic damage.

With the emphasis now on vessel recanalization, but facing issues of time and access, neuroprotective adjunctive strategies are more important than ever before. However, neuroprotective agents for ischemic stroke have been studied extensively, with notable failures in clinical trials [28]. Reasons for failure included lack of translatability in pre-clinical testing (stroke models and laboratory animals that fail to represent actual stroke patients and their underlying comorbidities, age and gender differences as discussed in greater detail below), extended time windows of administration, mismatching of stroke severity/subtype in pre-clinical and clinical conditions, clinical trial design, lack of study on combinational drug therapies, and the heterogeneity of target populations in studies. Current strategies under study include a focus on distinct time points: pre-hospital, intravenous administration coupled with tPA +/− thrombectomy during acute treatment, intra-arterial administration during thrombectomy, and systemic administration after consideration of initial recanalization therapy [29–31]. In designing and evaluating novel therapeutics, researchers should be mindful of these potential windows, designing pre-clinical studies to mirror the target window in this paradigm.

Ample windows for therapeutic development also exist in hemorrhagic stroke. Intracranial hemorrhage (ICH) is a severely disabling stroke subtype, where data for aggressive intervention are relatively lacking compared to subarachnoid hemorrhage and ischemic stroke. Treatments continue to be evaluated that are aimed at the major contributors to brain injury: edema, oxidative stress, inflammation, and potential for additional bleeding [32]. Furthermore, secondary injury occurs within 24 h, as iron and heme induce inflammation, reactive oxygen species, and glutamate release, and inhibit DNA repair [33]. Compounds currently under study include such drugs as deferoxamine, minocycline, statins, celecoxib, desmopressin, and conivaptan. These drugs each addresses some aspect of the pathology noted in the mechanisms above [32, 34, 35]. Similar to stroke and thrombectomy in the past, advances in ICH treatment have been stalled by a lack of significant positive data supporting evacuation/removal of the hematoma. Currently active trials are evaluating the utility of minimally invasive strategies for clot removal such as catheter-based clot thrombolysis (MISTIE) and port-based microsurgical removal (ENRICH) [36, 37]. In that vein, additional opportunities exist for translational research in ultra-early treatment strategies (prior to secondary injury), combining surgical evacuation with neuroprotective/neuroreparative strategies, clot-directed neuroprotection, and systems of care development.

Aneurysmal subarachnoid hemorrhage and its feared consequence, vasospasm and related ischemic injury, also offer opportunities for translational research. Despite continued advancements in devices used to repair the aneurysms themselves, relatively little clinically demonstrated research has changed the treatment of cerebral vasospasm. Common treatments include nimodipine in the pre-vasospasm interval to reduce injury, induced hypertension to increase cerebral perfusion, and endovascular strategies. Those include transluminal balloon angioplasty and intra-arterial drug administration; typical agents include verapamil, papaverine, nimodipine, and milrinone. Current areas of study include nitric oxide donors, endothelin-1 antagonists, low-dose heparin, and intrathecal agents; the NEWTON2 trial is currently underway to evaluate intraventricular sustained-release nimodipine for SAH [38]. Opportunities exist for developing sustained-release spasmolytics, as well as intra-arterial neuroprotectants that could be injected during the time of standard intra-arterial treatments for vasospasm.

### Animal Models—The Stroke Researcher’s Workhorse (Presented by S. Thomas Carmichael and Miguel Perez-Pinzon)

Animal models represent a research workhorse for developing novel therapeutics in stroke and cerebrovascular disease. The most widely recognized is the MCA occlusion model in rodents. This involves a surgical neck dissection with isolation of the carotid bifurcation; using the external carotid artery (ECA) for access, a blunted suture is advanced through the internal carotid artery (ICA) to occlude (permanently or transiently) the ICA bifurcation intracranially. This model yields a large vessel-type occlusion, similar to an M1 occlusion in humans, with hemispheric involvement of both cortex and deep structure [39, 40]. Variations on this model exist, allowing the occlusion to be tailored to create different-sized infarcts with either permanent or transient occlusion [41, 42]. The photothrombotic model provides a focal cortical infarct, which can mimic distal small vessel infarcts in humans, except that in the model all vessels are thrombosed and just not arteries [43]. For hemorrhagic stroke, multiple models exist. For basal ganglia ICH, models include stereotactic autologous blood injection, stereotactic collagenase injection, and stereotactic striatal balloon inflation [44, 45]. For SAH and vasospasm, common models include cisterna magna autologous blood injection and ICA vessel perforation [44]. This model is similar to the MCAO model, but uses a sharp-tip filament to puncture the ICA [46].

The selection of the type of animals plays an important role in model translation to human disease. Rodents represent the foundation model for most animal stroke research, while large animal studies are often used for more confirmatory experiments in novel therapeutics. Furthermore, while therapeutic research may often begin with single-gender, young, wild-type normal animals, experimental planning should expand into aged, comorbid animals of both genders [47–49]. To address the importance of translatability to human disease, multiple working groups have published recommendations for pre-clinical animal research in stroke therapeutics. These include such groups as the Stroke Therapy Academic Industry Roundtable (STAIR), Stem Cell Therapies as an Emerging Paradigm in Stroke (STEPS), and Stroke Recovery and Rehabilitation Roundtable (SRRR) [6, 7, 50–53]. Common recommendations made include the experimental evaluation of both genders, aged animals, comorbid animals, multiple-laboratory collaboration for reproducibility, long-term behavioral evaluations, use of biomarkers when appropriate to measure treatment effect, and dose-response curve evaluations. Additionally, as previously mentioned, time windows for interventions should reflect important clinical timelines in current treatment paradigms, and treatment effects should be demonstrated to be causative from mechanism to effect rather than correlative whenever possible.

In terms of clinical outcome assessments in stroke, there should be rigor in behavioral and cognitive assessments. Cognitive assessments such as the Barnes Maze represent spatial memory tests that are species-appropriate, and allow for recording and evaluation of multiple variables such as distance, latency, error rate, and search strategy [54]. Other valuable tools include contextual fear conditioning, Y or T mazes to test executive function, and zero mazes and sweetened water for anxiety/depression. While performing all such tests in every experiment is prohibitive, including more than one test in an experimental cohort provides both confirmation of pathology and increased opportunity to demonstrate treatment signal.

Finally, animal models offer an opportunity to screen potential therapeutic targets. While positive findings are encouraging and supportive of clinical trials, negative data can be just as informative. Indeed, the rigor needed to disprove a hypothesis is, in many ways, more significant. In cases of small *N* numbers, negative results may likely represent a beta error. As such, exhaustive evaluations that result in uniformly negative data should be celebrated. Publication of well-designed experiments with negative results should be a priority as it informs the scientific space [55, 56].

### Animal Models—Diamonds in the Rough (Presented by Halina Offner and Lauren Sansing)

#### Do Animal Trials Translate to Human Subjects?

**Fig. 1:**
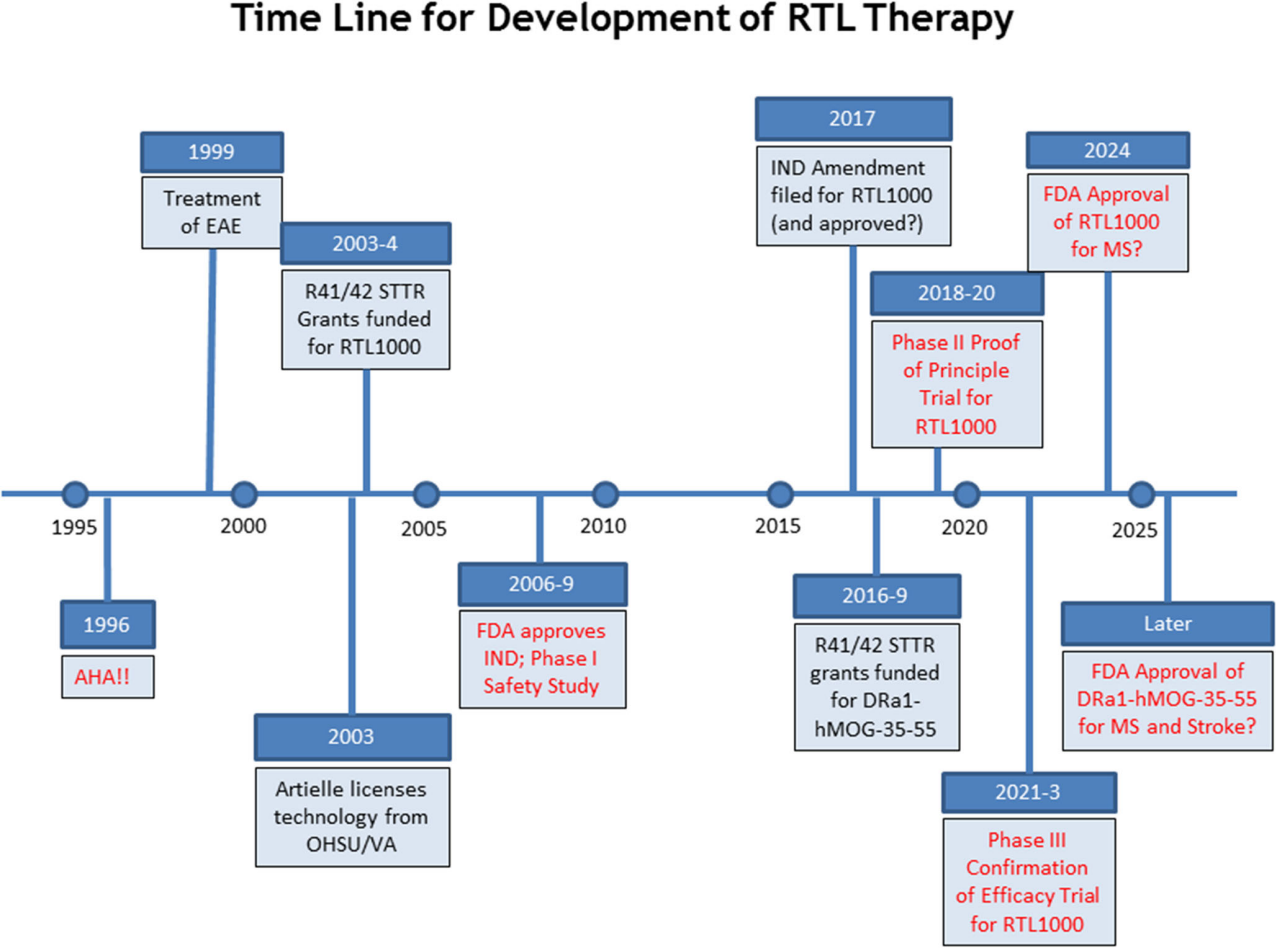
Timeline of the development of the RTL1000 for treatment in multiple sclerosis and stroke

Development of a novel therapeutic is a protracted process from animal models to a treatment for humans. Recombinant T cell receptor ligands (RTL) were presented as an example of drug development, which was first devised for treatment of multiple sclerosis (MS) and now has shown efficacy in rodent models of stroke [57]. The RTL is comprised of domains from MHC class II tethered to a myelin antigenic peptide, which inhibits activation of both monocytes and T cells. Monocyte activation is blocked by RTL1000 by competitively displacing macrophage migration inhibitory factor (MIF) binding to the CD74 receptor. A single dose of RTL1000 effectively suppresses symptoms in a mouse model of MS even after the onset of clinical signs. Delayed multiple daily doses were effective in reducing tissue damage in a chronic progressive MS-like disease model at least partially through downregulation of CD74 expression on monocytes. A phase 1 clinical trial was completed demonstrating no exacerbation of disease and CD74 as a key biomarker for MS. As shown by Fig. 1, this drug development began in 1996 and requires decades from basic research using relevant animal models to clinical trials. The business grant mechanism from NIH, STTR/SBIR, is important to support and facilitate drug development towards clinical use.

Since RTL1000 is anti-inflammatory, and blocks cell migration into the brain, it has been applied to treating experimental stroke. Mouse models do provide enough similarities to the human immune response to stroke for translational studies [58]. To demonstrate the adaptive immune system’s detrimental role in stroke, splenocytes sensitized to myelin oligodendrocyte protein were administered to SCID mice with transient MCAO [59]. These mice had larger infarcts and poorer neurological outcomes. Since RTL1000 interferes with the adaptive response, this agent was used to treat male, female, and middle-aged male mice after MCAO [60]. The efficacy of RTL1000 was not dependent on sex or age of the mice and was effective in other stroke models as well as in combination with tPA. Additionally, this treatment increased the number of splenocytes while decreasing immune cells in the injured hemisphere. These studies were in accordance with the updated STAIR recommendations that state studies should include both sexes and aged animals [61] and suggest that this approach could be successful in treating human stroke patients.

One problem with RTL1000 is that its beta-1 domain must match the recipient patient’s MHC II. HLA-DRα1-MOG35-55 is a novel RTL that does not contain the beta-1 domain. DRα1-MOG-35-55 was found to be neuroprotective in ischemic stroke in male and female mice [62]. DRa1-MOG-35-55 treatment reduces CD74 expression in ischemic brain after MCAO. The expression of CD74 is upregulated after ischemic stroke and is associated with stroke severity. Moreover, CD74 changes in blood may be useful as a biomarker for stroke.

In summary, a successful pharmaceutical approach for one neurological pathology may also be utilized to other insults or diseases of the brain. The development of a new pharmaceutical for treatment of brain diseases or injury requires many years until they are readily available to the patient. The NIH does provide grant mechanisms such as STTR/SBIR to expedite the process. However, the pre-clinical studies must include both sexes and aged rodents to enhance the probability of translation to the human patient.

#### Successes in Translation and Attempts at Reverse Translation

While the conference has emphasized the shortcomings of pre-clinical stroke research, there have been success stories, such as intravenous tPA for acute stroke, flow diversion for intracranial aneurysms, and tPA used for intraventricular hemorrhage. The use of tPA within a 3-h window from last known normal improves outcomes 33% of the time while only worsening outcomes 3% of the time [63]. The first publication on tPA described its use in a rabbit embolic stroke model [64]. This rabbit model was used successfully to determine dose and adverse effects in the development of tPA for human patients [65]. Intracranial aneurysms are balloon-like dilations of cerebral arteries that represent 3% of all strokes [66]. Models using dogs and rabbits were utilized to perfect intravascular flow diversion devices to treat aneurysms [67–69]. The first intraventricular delivery of a thrombolytic used urokinase in a dog model [70]. This pre-clinical research led to CLEAR III clinical trials [71] and MISTIE III [72]. These studies demonstrated that animal models should be developed to answer the specific question and employ experimental rigor (ex: randomization, blinding). Most importantly, insights from human data and the causes of patient poor outcomes must be taken into account to improve the animal models to be relevant to the clinical questions.

Importantly, human data should be utilized to fuel research in animal models for mechanistic studies. This strategy has proven fruitful in identifying granulocyte and monocyte phenotypes and corresponding molecular signaling in response to intracranial hemorrhage (ICH). In these studies, blood and hematoma samples from human ICH patients were used to identify subpopulations of neutrophils and monocytes reacting to the injury [73]. Over time after ICH, monocyte phenotype changed from an inflammatory to a reparative one [74]. Specific molecular pathways were then identified in mouse models and further verified in human patients. From this reverse translational work, several key signaling molecules have been identified that could lead to novel therapeutic approaches to hemorrhagic stroke.

**Fig. 2:**
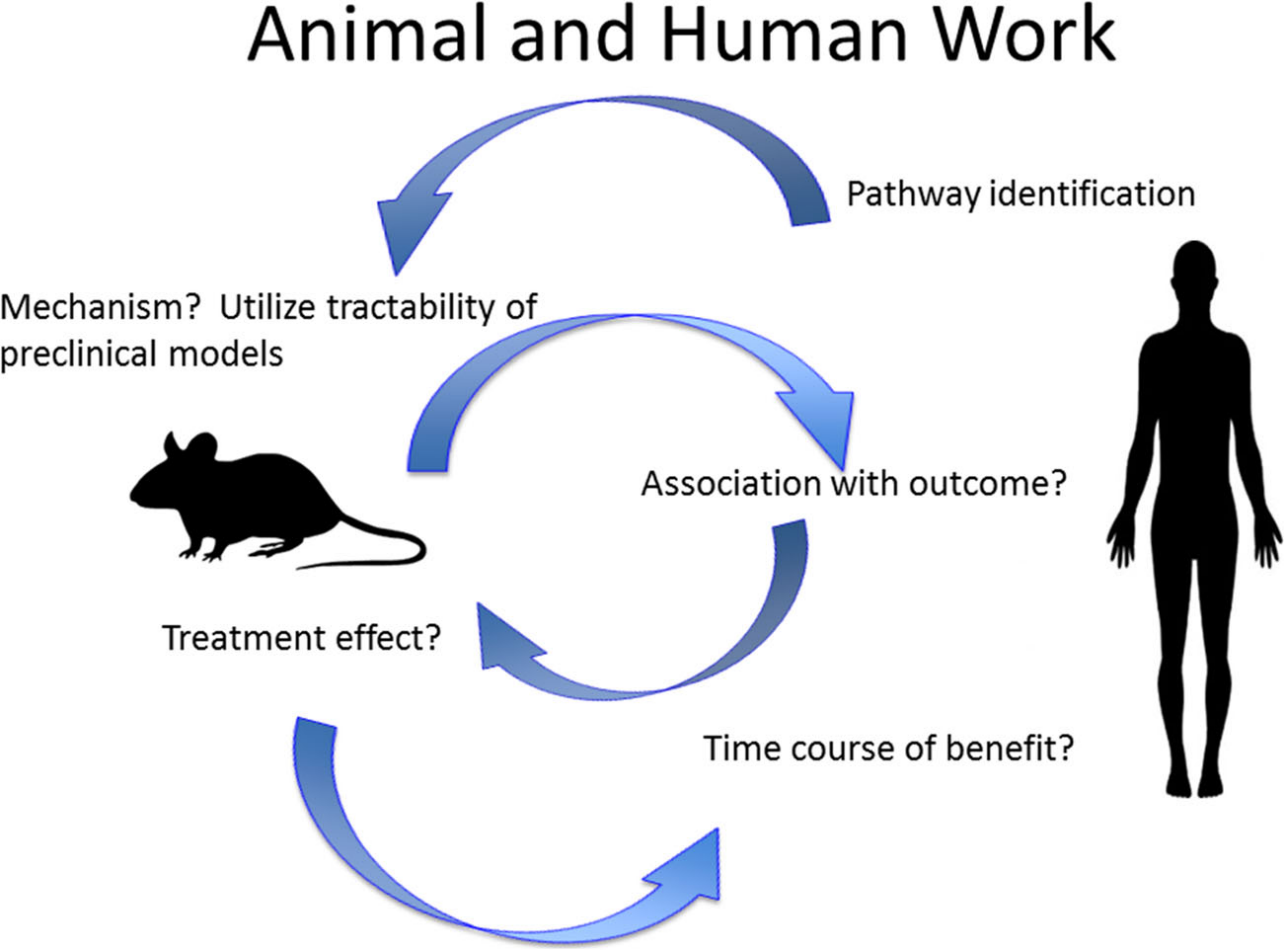
Reverse translation: back and forth research between human and animal studies

Despite an overall lack of translational success in stroke research, animal models did lead to the development of tPA and intravascular stent devices to improve outcomes in human stroke patients. However, more can be accomplished to perfect animal models through reverse translation, as schematically represented in Fig. 2. Observations in humans can be tested in animal models to mechanistically elucidate cellular and molecular pathways that can then be verified in the human condition.

### Grantsmanship for Translational Research (Presented by Francesca Bosetti)

The National Institute of Neurological disorders and Stroke (NINDS) supports the full spectrum of basic, translational, and clinical research in order to seek fundamental knowledge about the brain and nervous system and to use that knowledge to reduce the burden of neurological disease. NINDS supports research resources and technical advances that catalyze new therapeutic interventions for neurological disorders and is committed to training the next generation of scientists and supporting the increase of underrepresented groups in the growing field of neuroscience research, through a variety of individual and institutional training programs that incorporate dedicated mentorship (https://www.ninds.nih.gov/Funding/Training-Career-Development).

The mission of NINDS Division of Translational Research (DTR) is to facilitate the pre-clinical discovery and the development of new therapeutic interventions for neurological disorders. NINDS supported the body of research that led to current thrombolytic and endovascular therapies used in stroke and is actively supporting the discovery and development of new treatments for ischemic and hemorrhagic stroke. The NINDS Division of Translational Research offers several grant programs that nurture early-stage therapy development to Phases 1 and 2 clinical trials (https://www.ninds.nih.gov/Current-Research/Research-Funded-NINDS/Translational-Research). These programs are milestone-driven and offer multiple entry points in the therapeutic development pipeline. Small business innovation research/small business technology transfer (SBIR/STTR) grants are additional available mechanisms to support therapeutics, diagnostics, and tools for research and can include basic research, translational research, and early-stage clinical trials.

In November 2016, the NINDS-sponsored workshop “Translational Stroke Research: Vision and Opportunities” produced recommendations [10] to align pre-clinical outcome measures with phase II human outcome measures including cognitive outcomes [10], and use animal models that adequately model human strokes. Problems in translational stroke research include pre-clinical endpoints that do not reflect clinical outcome, lack of good lab practices to reduce bias, and a general lack of enthusiasm for publishing negative findings. On the clinical side, drugs may not be tested at the right time, duration, or dose, inclusion criteria may be too broad, and the common clinical outcome measure, the modified Rankin Score (mRS) scale, has clear limitations in the assessment of cognitive function and recovery of function. As stated above, to improve translation of novel stroke therapeutics, experimental animal models need to recapitulate human stroke, and validate pre-clinical target to make sure they are applicable for the human population. One of the recommendations indicated the potential value of multicenter networks, with centralized randomization and data management, for pre-clinical testing of late-phase promising therapies.

### Current and Future Debates in Translational Stroke Research (Moderated by Cenk Ayata)

The format was an open discussion that encouraged interaction between the panelists and the attendees. While several issues were deliberated, most of the dialog centered on the team-building approach to connect clinicians and basic scientists. One issue that separates clinical and basic scientists is that they are often physically housed in different areas of a medical center/university, which creates a natural barrier to inertia in collaboration. There is a need to incentivize cross-talk. Researchers should be encouraged to attend clinical seminars and meetings, such as grand rounds, to interact with clinicians as well as to develop an understanding of the clinical environment. Conversely, clinicians are incentivized to see patients and provide income for the hospital so it is difficult to allocate time for research. While “protected time” is important, a more novel approach may be to organize translational research efforts along service-line models from a high-level administrative standpoint.

## Conclusions

Translation of stroke therapeutics from bench to bedside remains a major challenge. One barrier is the relative heterogeneity of stroke in the human condition compared to animal models. Improving animal models to closely mimic the specific stroke condition under study for targeted therapeutics is vital. Second, fostering continuous cross-talk between clinicians and basic researchers is a priority. Service-line grand rounds/seminars and administratively supported research time are vital. Finally, bringing researchers into the clinical spaces is important; reverse translation informs the animal models for testing new therapeutics. Through such efforts, the next phase of stroke therapies can increase their chance to succeed.
